# Functional fermented foods in public health nutrition: key biomolecular mechanisms, gut microbiota interactions, and implications for metabolic disease prevention

**DOI:** 10.3389/fnut.2026.1818528

**Published:** 2026-06-23

**Authors:** Saleh A. Alsanie

**Affiliations:** 1 Department of Basic Health Sciences, College of Applied Medical Sciences, Qassim University, Buraydah, Saudi Arabia; 2 Department of Clinical Nutrition, Medical City, Qassim University, Buraydah, Saudi Arabia

**Keywords:** bioactive peptides, functional fermented foods, gut microbiota, metabolic disease prevention, probiotics, public health nutrition, short-chain fatty acids

## Abstract

Functional fermented foods are increasingly recognized in public health nutrition for their potential to reduce the burden of metabolic diseases through fermentation-derived bioactive biomolecules. Fermentation enhances the nutritional and functional properties of foods by generating peptides, short-chain fatty acids, organic acids, exopolysaccharides, enzymes, and transformed phytochemicals with antioxidant, anti-inflammatory, immunomodulatory, and metabolic regulatory activities. Despite growing interest, the biomolecular pathways linking these compounds to metabolic health, particularly through gut microbiota modulation, remain insufficiently clarified. This review focuses on representative functional fermented foods such as dairy, cereal, legume, vegetable, tea, and traditional mixed fermented foods and key fermentation-derived biomolecules, including bioactive peptides, short-chain fatty acids, polyphenols, exopolysaccharides and organic acids, with emphasis on their mechanistic roles in gut microbiota modulation and metabolic disease prevention. By linking biomolecular mechanisms with public health outcomes, it positions functional fermented foods as promising, sustainable tools for metabolic disease prevention and health promotion.

## Graphical Abstract

**Figure:**
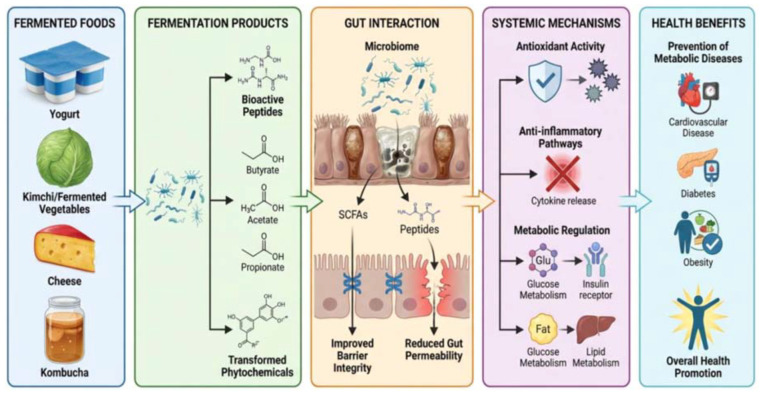

## 1 Introduction

The global landscape of the public health is presently dominated by the staggering increase in the incidence of non-communicable diseases (NCDs) with metabolic disorders accounting for the largest share of this burden. Obesity, type 2 diabetes (T2D), cardiovascular disease (CVD), have become the main determinants of reduced lifespan and wellbeing in developed and developing countries. A major cause of this epidemiological change is generally the dietary shift known as the nutrition transition, which involves the adoption of Westernized dishes with a high amount of ultra-processed foods, refined sugars and saturated fats and increasingly sedentary lifestyles. These metabolic states are not simply the product of caloric imbalance but lie at the base of chronic and low grade systemic inflammation and endotoxemic metabolism (1). Such systemic pathologies are often associated with gut microbial dysbiosis, refers to an imbalance in microbial composition and function characterized by reduced microbial diversity, depletion of beneficial taxa including *Akkermansia* and *Bifidobacterium*, and subsequent intestinal ecosystem imbalance that impairs host-microbe signaling and metabolic homeostasis (2).

The intestinal microbiota is considered as key metabolic organ and it serves as a bridge between nutrients in food and intestinal physiology. The strongest modulator of microbial composition and activity is diet, which affects the metabolites production that control the epithelial barrier integrity, immune response, and metabolism of glucose and lipids. The loss of microbial variety and the growth of pro-inflammatory taxa in metabolic illnesses result in a permeable intestinal wall enabling the translocation of lipopolysaccharides (LPS) in the bloodstream leading to chronic inflammation (3). So, the preventive nutrition approaches which address the gut environment to counter metabolic imbalance are required. This has led to a resurgent interest in functional fermented foods as precision tools in public health nutrition. Contrary to common processed foods, fermented foods are living media to provide beneficial microorganisms (probiotics), as well as a complex network of fermentation-derived bioactive compounds (postbiotics) (4).

Fermentation is a food conversion process whereby food components are degraded, through the action of microorganisms such as lactic acid bacteria (LAB), to produce a enriched nutritional profile nutrients. The health advantages of fermented foods are not necessarily associated with the survival of live cultures but are more likely to be related to the production of certain biomolecules. Dairy or plant-based proteins that are digested by microorganisms during fermentation produce bioactive peptides which induce alteration of host gene expression concerning glucose signaling and anti-inflammatory signaling (5). Moreover, fermentation of carbohydrates and fibers leads to the formation of short-chain fatty acids (SCFAs), i.e., acetate, propionate, and butyrate. These SCFAs are signaling molecules, which interact with host receptors and regulate appetite, enhance insulin sensitivity, and enhance the intestinal barrier integrity. Some LAB strains also form exopolysaccharides (EPS), which connect to the food physical structure and react with the mucosal immune system of the host to generate metabolic dysfunction inflammatory drivers (6).

Despite this, fermented foods have a promising therapeutic potential, there are still substantial knowledge gaps that make it impossible to formulate ultimate clinical guidelines. The consumption of fermented dairy and its derivatives has been associated with a lower risk of T2D and cardiometabolic indicators; however, the biomolecular mechanisms underpinning the effect have not been fully determined (7). The current studies usually did not differentiate the effects of the live microbes and their metabolites formed during the fermentation process. Moreover, the interactions between these fermentation-derived biomolecules and the host’s existing, highly personalized resident microbiota and the effect of the food matrix on these interactions, have not yet been adequately characterized. Also, there is lack of long-term, standardized human clinical trials, which use multi-omics methods to prove cause-effect relationships between selective fermented food intake, microbiota changes, and clinical metabolic performance (8).

This review aims to synthesize current evidence on the role of functional fermented foods in the prevention and management of metabolic diseases. This review focuses on core bioactive molecules generated during fermentation, including bioactive peptides, short-chain fatty acids (SCFAs), exopolysaccharides (EPS), and organic acids, as well as representative fermented food matrices encompassing dairy, cereal, vegetable, legume, and fermented tea products. By exploring the complex interplay between fermentation-derived biomolecules (peptides, SCFAs, and EPS), the gut microbiota, and host metabolic signaling, this article seeks to bridge the gap between traditional dietary practices and modern molecular nutrition. The scope includes an analysis of both dairy and plant-based fermented matrices, evaluating their efficacy in modulating cardiometabolic risk factors. Ultimately, this review provides a framework for future research and public health strategies centered on precision fermented nutrition as a cornerstone of metabolic disease prevention.

## 2 Literature search methodology

A comprehensive literature search was conducted using electronic databases including PubMed, Scopus, Web of Science, and Google Scholar for studies published between 2000 and 2026. Keywords and combinations of terms such as “functional fermented foods,” “gut microbiota,” “bioactive peptides,” “short-chain fatty acids,” “metabolic syndrome,” “type 2 diabetes,” “obesity,” and “public health nutrition” were used. Original research articles, clinical trials, systematic reviews, and meta-analyses published in English were prioritized. Studies focusing on fermentation-derived biomolecules, microbiota interactions, and metabolic health outcomes were included, while unrelated studies and non-peer-reviewed reports were excluded. Reference lists of selected articles were also manually screened to identify additional relevant publications.

## 3 Functional fermented foods in public health nutrition

### 3.1 Functional fermented foods

Functional fermented foods represent an important interface between traditional food processing and modern nutritional science, and are defined by the fact that they possess health benefits beyond simple satiety and the provision of macro and micronutrients. Although traditional fermented foods are more generally described as products that are produced by means of controlled microbial growth and enzymatic transformation of food components, the term “functional” specifically suggests that the products are designed or identified to provide specific physiological benefits (9). This difference is central to the field of public health nutrition though traditional fermentation often depends on spontaneous microbial communities to preserve and add flavor to food, functional fermented foods are often linked with the addition of specific probiotic cultures or an intentional increase in bioactive metabolites (10). The conceptual basis of these foods is that the products are designed to serve as vehicles to deliver live, viable microorganisms at high levels, i.e., in the range of 10^7^ to 10^9^ colony-forming units per gram or milliliter, which may be necessary to produce biologically meaningful effects in the human GIT. This concentration guarantees that enough numbers of microbes can survive the large quantities of acid present in the stomach to engage in the interaction with the immune system and resident host microbiota (11).

**FIGURE 1:**
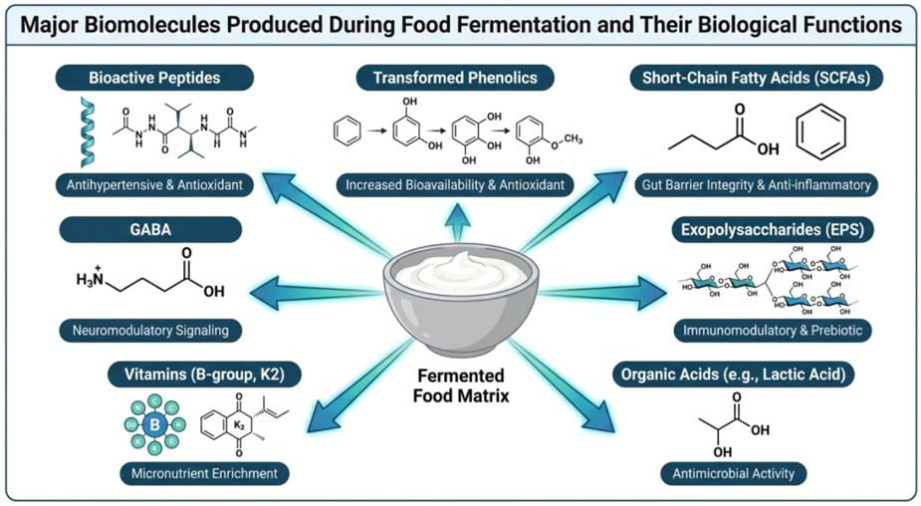
Major biomolecules produced during fermentation and their biological function.

In addition to having live cultures, functional fermented foods also includes a wide range of biomolecular properties that are produced during fermentation. It consist of bioactive peptides, microbial EPS and SCFAs that get released during proteolysis. These compounds act as molecular signals to modulate host metabolic pathways as shown in Figure 1. For example, fermented dairy bioactive peptides have been widely investigated on their antihypertensive and insulin-sensitizing effects, which gives them a mechanistic pathway of application in dietary interventions focused on prevention of T2D and CVD (12). Moreover, LAB generate microbial EPS that are known to have prebiotic-like properties, which increase the growth of native gut bacteria and improve the metabolism rate of glucose. Such foods are now being perceived not as food choices but as preventive measures in the control of NCDs in the context of public health. Functional fermented foods mediate systemic inflammation and metabolic homeostasis by modulating the microbiota present in the gut and enhancing the intestinal barrier (13). The main issue is still the absence of a universally accepted regulatory standard. The community now reflects on the significance of strain-specific characterization, and the characterization of a particular set of fermentation-derived bioactives as compared to a single, unitary definition (14). Amid the increasing emphasis on the food as medicine framework in public health nutrition, functional fermented foods have emerged as a continuously evolving research field. These products are transitioning from traditional food practices to evidence-based applications to reduce the risk of metabolic disorders (15).

### 3.2 Classification of fermented foods

Functional fermented food can be categorized broadly by its raw materials, which dictate that unique microbial ecology and specific bioactive products they deliver to the host. The most significant fermented foods in the context of public health nutrition are dairy-based fermented foods, i. e. the yogurt and kefir fermented foods. These products serve as primary delivery systems for well-characterized probiotic strains, including *Lactobacillus* and *Bifidobacterium*. Upon transit through the gastrointestinal tract, these microbes regulate gut microbiota composition (16). In addition to live cultures, dairy fermentation results into bioactive peptides, including casomorphins and lactokinins, that have antihypertensive, immunomodulatory, and opioid-like effects on the molecular level. These metabolites play a crucial role in managing cardiometabolic risks by inhibiting angiotensin-converting enzymes and improving insulin sensitivity (1).

Fermentations of cereals and grains such as sourdough bread, idli, and, traditional African porridges such as ogi contribute to nutritiousness of staple crops. This classification is especially significant in its capability to eliminate antinutrients, which are naturally occurring compounds that can hinder nutrient absorption, such as phytic acid, and subsequently boost the bioavailability of essential minerals like iron, zinc, and magnesium. The microbial activity in grains synthesizes B-vitamins such as folate, riboflavin and form EPS. These bioactive components function as prebiotics, which selectively support beneficial gut bacteria proliferation and metabolism, and decrease the glycemic index of final foods (17). Fermented vegetables and legumes, such as kimchi, sauerkraut, and tempeh, are known to contain a high amount of fiber, as well as phytochemicals. In the fermentation process, the complex polyphenols are converted by the microbial enzymes to the more bioavailable compounds, such as aglycones, which have very strong antioxidant and anti-inflammatory properties (11). Moreover, organic acid production such as lactic and acetic acid suppress pathogens and maintain the healthy intestinal pH. Fermented legumes such as miso and natto are also remarkable as one of the sources of vitamin K2, bioactive peptides that promote bone and cardiovascular health (12).

Fermented teas represent another globally important category of functional fermented foods, including dark tea, kombucha, and post-fermented tea products. These matrices are fermented via complex consortia of fungi, yeasts, and lactic acid bacteria, leading to the biotransformation of tea polyphenols into unique bioactive compounds such as theabrownins, theaflavins, and theasinensins (18). These metabolites improve glucose and lipid metabolism by activating AMPK/PI3K/Akt signaling and enhancing GLUT4 translocation, thus alleviating insulin resistance and hepatic steatosis. Fermented tea also enriches *Bacteroides*, *Alistipes*, and SCFA-producing bacteria, reinforcing gut barrier function and reducing metabolic endotoxemia. As widely consumed products, fermented teas provide consistent metabolic benefits that complement dairy, cereal, and plant-based fermented foods (19).

Meat and fish fermentations not only believed to preserve and bring flavors to meat but are also gaining more recognition due to their possible functionality. In fermented sausages, as well as traditional fish sausages, certain lactic acid communities assist in degradation of muscular proteins into bioactive peptides and free amino acids that could control systemic antioxidant defense and metabolic regulation (20). Even though these products may be taken in smaller quantities, the dietary variety and provision of particular metabolites produced by the microorganism cannot be ignored in the real-life scenario of the given population. The heterogeneity of such classifications reflects the fact that the functionality of fermented foods is not a homogenous phenomenon, but rather a synergistic product of the nutritional properties of the substrate and particular microbial changes that occur during the fermentation process (14). Compared to general health approaches, personalized nutrition has become a more prominent trend in population healthcare, necessitating the elucidation of these substrate-specific mechanisms underlying the focused prevention of metabolic illness and facilitation of nutritional resilience at the global scale (21). The classification of functional fermented foods across different food matrices is summarized in Table 1.

**TABLE 1:** Classification of functional fermented foods across different food matrices.

Matrix	Representative examples	Dominant microbial consortia	Traditional origin	References
Dairy	Kefir, yogurt, koumiss, bryndza	Lactic acid bacteria (LAB) (*Lactobacillus*, *Lactococcus*, *Streptococcus*); yeasts (*Saccharomyces*, *Kluyveromyces*)	Caucasus, Central Asia, Europe	(22, 23)
Cereal and grains	Sourdough, tarhana, boza, kvass	LAB (*Lactobacillus* spp.); yeasts (*Saccharomyces cerevisiae*)	Europe, Middle East, Africa	(24, 25)
Vegetables and legumes	Kimchi, sauerkraut, tempeh, natto	LAB (*Leuconostoc*, *Pediococcus*, *Lactobacillus*); *Bacillus* (natto); molds (*Rhizopus* for tempeh)	East Asia, Southeast Asia, Europe, Indonesia	(26, 27)
Meat and fish	Fermented salami, cured fish, fish pastes	LAB (*Pediococcus*, *Lactobacillus*); mixed salt-tolerant consortia (*Staphylococcus*, *Micrococcus*)	Mediterranean, East and Southeast Asia	(13, 28, 29)

### 3.3 Role in traditional diets and population-level nutrition

The inclusion of fermented foods into human diets is a millennia-old and intercultural phenomenon that served as the basis of traditional nutrition systems all over the world. Traditionally, fermentation was highly appreciated due to its capability to save perishable materials like milk, vegetables and meats without refrigeration. However, current public health studies reveal that these customary dietary habits improve food security and exert a key regulatory role in sustaining population metabolic health (30). Since the Middle East and Eastern Europe began to consume yogurt and kefir, as well as East Asia began to consume kimchi, miso and tempeh, fermented foods have historically played an important share of the daily bioactive compound and live microorganism intake. Epidemiological data shows that populations consuming high amounts of fermented foods have lower NCD prevalence, especially diseases associated with metabolic disorders (1).

Fermented foods also play a role in the prevention of metabolic syndrome at population level as it regulate the risk factors that put people at risk. The regular intake of fermented dairy products, including yogurt, has a negative relationship with the occurrence of T2D and cardiovascular events (31). Not only dairy, fermented vegetable foods in traditional diets contain important fiber and phytochemical which are biotransformed by microbial activity to more effective anti-inflammatory and antioxidant action. This is especially true with respect to the phenomenon of the nutrition transition, in which the replacement of traditional fermented staple products by deeply processed, shelf-stable, and sterile modernized foods has been accompanied by an unprecedented increase in obesity and other associated metabolic conditions. This change in nutrition has resulted in a drop in the diversity of gut microbes, commonly referred to as the disappearing microbiota hypothesis, which is associated with the rise in chronic inflammatory conditions (32).

Moreover, the potential of fermented foods as cost-effective and culturally acceptable disease prevention interventions is of importance to their public health implications. Fermented foods have become the main source of quality protein and vitamins especially to many traditional communities particularly those with limited access to a wide range of food resources. Even the glycemic index of the staple grains and legumes can be lowered by the process of fermentation itself, making them better suited for people vulnerable to glucose intolerance. These foods deliver micronutrients with precise impacts on systemic wellbeing by promoting micronutrient bioavailability and generation of the metabolites such as SCFAs and bioactive peptides (33). The global epidemic of metabolic diseases needs to be checked by reintroducing traditional fermented foods into current dietary habits as more and more households adopt personalized and microbiota-based approaches to their health management. This paradigm acknowledges that the wellbeing of a population is inextricably coupled with the symbiotic connection of their diet with their gut microbiota and the millennia-old food-processing methods that have enabled the preservation of human health (34).

### 3.4 Sustainability, accessibility, and cultural relevance

Sustainability, accessibility and cultural relevance of functional fermented foods are the key determinants of their success as interventions in global population health, especially in resource constrained and diverse environments. Fermentation is intrinsically connected with sustainability as it is a low-energy, household-level form of preservation technology, which greatly minimizes the application of industrial cold chains and high-energy preservation systems. Fermentation is a microbial method of stabilizing perishable substrates, including dairy, vegetables, and cereals, by microbial acidification to extend their shelf life, reduce food loss, and manage resources and food supply (35). Traditional fermentation in West Africa can be optimized to process the surpluses of the millet crop so that it can add local value as well as reduce wastage, and this should be an effective substitute for energy-demanding modern processes (1). Moreover, the low infrastructure needed in fermentation makes it a secure food preservation system under climatic change and unstable energy supply contributing to long-term food security (36).

Both accessibility and affordability are also very important because fermented foods are commonly manufactured using locally sourced cheap raw materials thus readily available to low-income earners. The incorporation of domestically-produced fermented milk, including the Zambian Mabisi, into the diets of children would enhance the consumption of vital nutrients more efficiently than their non-fermented local counterparts (37). Fermented pulse-based products and cereals are low-cost functional ingredients that can be used to combat poverty and malnutrition in children through high-quality protein and greater mineral bioavailability, without expensive added supplements. Additionally, social enterprise types and community-based production workshops which are offered to underserved communities will give them the ability to implement their own probiotic-enhanced beverages as alternative to high-fructose corn as proposed in Brazil. This regionalized strategy means that functional nutrition does not remain a luxury, but rather an essential element of the daily foods of people who are the most at risk of metabolic and nutritional deficiencies (38).

Cultural relevance is an effective force toward the adoption and sustainability of functional fermented foods in healthcare systems. These foods have become part of the culinary heritage, ritualistic practices and social identities of ethnic communities in the global society and have resulted in high levels of consumer acceptance and transgenerational knowledge transfer. Since these products are already established, the nutrition interventions based on the enhancement of conventional customs face less cultural resistance than implementing new, artificial useful food products (39). Moreover, homemade fermented products are also produced and sold as livelihoods to small producers and women cooperatives, which enhance the household economies and the ability of communities to withstand, thereby increasing resilience (40). Based on the cultural relevance and economic opportunity of traditional fermentation, public health initiatives can generate sustainable dietary changes that are not only biologically effective but also become culturally relevant. Finally, the relationship among environmental sustainability, economic accessibility, and cultural continuity makes functional fermented foods invaluable remedies to reach an objective of global nutritional equity and reducing the metabolic disease risk (41).

## 4 Biomolecules generated during food fermentation

### 4.1 Bioactive peptides

Bioactive peptides are important active biomolecules produced during the fermentation of a dairy and a non-dairy substrates. These small chains of amino acids are naturally built in coded form into larger proteins, which are inactive until the process called enzymatic proteolysis sets them free. The major process of their formation occurs through the complex proteolytic system of LAB. In the process of fermentation, LAB use cell-envelope proteinases and intracellular peptidases to break complex proteins, such as caseins in milk or globulins in legumes, into smaller molecules. This process results in release of a great number of bioactive peptides that may survive through gastrointestinal digestion and have a systemic physiological impact on the host (42).

The preventive health-promoting capabilities of these peptides are directly applicable to metabolic diseases prevention. One of the best-known effects is their antihypertensive nature which occurs via angiotensin-converting enzyme (ACE) inhibition. Val-Pro-Pro (VPP) and Ile-Pro-Pro (IPP) are peptides that are naturally occurring ACE inhibitors in fermented milk and grains and facilitate healthy blood pressure (43). Additionally, peptides produced through the process of fermentation have antidiabetic effects because they prevent dipeptyl peptidase-IV (DPP-IV). These peptides increase insulin secretion and increase glucose homeostasis by extending the action of incretin hormones such as GLP-1, which is a dietary approach to insulin resistance management (44). Morever, these peptides have antioxidant and immunomodulatory properties. The reactive oxygen species (ROS) can also be scavenged by specific amino acid sequences which is important in the reduction of the chronic low-grade inflammation linked to obesity and metabolic syndrome. The biomolecules inhibit the pro-inflammatory cytokines, thereby providing a molecular connection between the traditional fermented foods and the systemic regulation of host metabolism (45).

### 4.2 Short-chain fatty acids and microbial metabolites

Short-chain fatty acids (SCFAs), especially acetate, propionate and butyrate, are the main biomolecular end-products of microbial fermentation, especially using non-digestible carbohydrates and dietary fibers as a substrate. These metabolites are important signaling molecules that mitigate the relationships between food consumption, microbial activity and host metabolic wellbeing (46). The most common SCFA in the systemic circulation is acetate, which is a substrate for the hepatic lipogenesis and of the cholesterol synthesis, and which has an effect on the central nervous system, encouraging satiety and energy expenditure control. The liver is the main site of propionate metabolism and is known to be involved in gluconeogenesis and has even been indicated to suppress the *de novo* synthesis of cholesterol thus helping to improve lipid profiles (47).

Butyrate is the preferred energy source of colonocytes and plays a role in intestinal barrier integrity maintenance. It does this by blocking histone deacetylases (HDACs) and exerting a potent anti-inflammatory effect through up-regulating tight junction proteins and leading to a reduction of the translocation of pro-inflammatory LPS systemically (48). The physiological and metabolic significance of these SCFAs is facilitated by their action on certain G-protein coupled receptors, mostly GPR41 (FFAR3) and GPR43 (FFAR2) highly expressed in enteroendocrine cells, immune cells, and adipocytes. GLP-1 (glucagon-like peptide-1) and peptide YY (PYY) are anorexigenic gut hormones activated by SCFAs, the triggering of which is required in glucose homeostasis and appetite regulation (49). This incretin action promotes insulin release in pancreatic beta cells and decelerates gastric emptying, which offers a solid mechanistic pathway for preventing T2D and obesity. Moreover, SCFAs are important in the regulation of the gut-immune-metabolic axis. For instance, buckwheat-derived SCFAs and LAB metabolites have been reported to beneficially affect the metabolic syndrome phenotype by reorganizing the gut microbiota and lowering systemic inflammation (50). Most of the mechanistic evidence is based on preclinical models, but the incorporation of SCFA-enriched functional fermented foods in the diet is also becoming accepted as a scalable approach to public health. These foods do not just naturally contain SCFAs but also contain the microbial consortia needed to maintain SCFAs *in vivo*, thereby reinforcing the gut barrier and reducing the metabolic endotoxemia of high-fat, low-fiber modern diets (51).

### 4.3 Organic acids and fermentation-derived metabolites

Food fermentation is a biological manufacturing facility to produce a wide range of organic acids and metabolites, also informally known as postbiotics, which have significant effects on host metabolic health. In both plant and dairy matrices, LAB and acetic acid bacteria are the key agents which convert simple carbohydrates in to lactic and acetic acid. This acidification not only extend the life of the food it but also improves the bioaccessibility of the phytochemicals and alters the starch structure, which is important in glycemic management (52). On top of these basic acids, there are other metabolites such as the succinate, which are crucial intermediates during microbial cross-feeding. Although succinate was considered to be a byproduct in the past, recent findings showed that it is an extra signaling molecule, which balances the luminal environment and alters the gut microbiota into a more resilient community structure (53).

The biomolecular pathways that mediate the health benefits of organic acids act through both local and systemic pathways. Acetate is a SCFA that is a ligand for G protein-coupled receptors (GPCRs), GPR41 and GPR43. Stimulation of these receptors in the adipose tissue and small intestine initiates the release of incretin hormones such as glucagon-like peptide-1 (GLP-1) and peptide YY (PYY), which plays a role in glucose homeostasis and in the regulation of appetite (54). Moreover, SCFAs inhibit the process of hepatic lipogenesis by suppressing SREBP-1c and enhance hepatic β-oxidation in mitochondria by utilizing AMPK-activated signaling, which decreases the amount of ectopic fat deposition and enhances lipid phenotypes in models of metabolic disease (55). The SUCNR1 receptor also signals in response to succinate, which has been found to be a major controller of intestine barrier function; it enhances goblet cell differentiation and anti-inflammatory effects caused by a high-fat diet, effectively overturning barrier dysfunction (53).

Kimchi, kombucha, and natto are examples of fermented foods. The interaction of lactic acid and polyphenols enhances the bioaccessibility of the aglycones in kimchi that inhibit the activity of both alpha- amylase and alpha- glycosidase, thereby delaying postprandial glucose absorption. Equally, the high acetic acid content of kombucha and traditional sourdough may decrease gastroduodenal tonsillary emptying and glycemic response (56). These changes in metabolism are commonly accompanied by changes in the metabolism of the gut microbiota, in which fermentation-derived metabolites promote growth of such beneficial species as *Akkermansia muciniphila*, which further strengthens the gut-metabolic axis (57). Together, these organic acids and products of fermentation contribute to a multi-targeted strategy for preventing the occurrence of metabolic diseases through increased glycemic regulation, strengthened lipid metabolism, and systemic inflammation linked to strong gut-microbiota interactions (58).

### 4.4 Exopolysaccharides and microbial polysaccharides

Exopolysaccharides (EPS) are versatile multifaceted microbial polymers produced by a wide range of microorganisms during population fermentation, particularly by LAB including *Lactiplantibacillus plantarum*, *Streptococcus thermophilus* and *Leuconostoc* species. Structurally, these polysaccharides can be further divided into homopolysaccharides, which are made of one type of monosaccharide (usually glucose or fructose) and heteropolysaccharides containing repeating units of two or more types of sugars, including glucose, galactose, and rhamnose (59). Recent structural characterization of *L. plantarum* isolated from traditional fermented sauerkraut EPS reported heteropolymeric chains with high molecular weights of more than 10^6^ Da, with flexible conformations and pseudoplastic rheological behavior (60). Conversely, some types of *Liquorilactobacillus mali* have been found to form dextran-like homopolysaccharides containing different α-(1→6) backbones and α-(1→3) side chains that have a significant impact on their physical properties and biological interactions (61).

The functions of EPS in the field of public health nutrition are complex, both as a techno-functional advantage in food systems and as a direct health-promoting advantage in the host. EPS can be used as natural bio-thickeners and stabilizers in fermented dairy and vegetable products, where the components enhance viscosity and reduce syneresis as well as provide cryoprotection to probiotic cultures (62). In addition to these structural functions, EPS serve as powerful prebiotics by selectively stimulating the proliferation of beneficial gut flora, e.g., *Bifidobacteria*, and allowing them to ferment into SCFAs. Such microbial polysaccharides also have a strong immunomodulatory capacity as they can activate macrophages and trigger the release of anti-inflammatory cytokines such as IL-10 and regulatory pro-inflammatory indicators (63). Moreover, LAB-derived EPS also have antioxidant and cholesterol-lowering effects; in particular, the radical-scavenging activity and the ability to prevent cholesterol uptake in *in vitro* models was reported in those isolated from *Lactiplantibacillus pentosus*. These polymers have structural diversity, such as molecular weight and branched distributions, which are key factor defining their metabolic effect, determining their capacity to alter glycemic reactions and reinforce gut barrier strength (64). Consequently, EPS are not perceived solely as functional components that promote food quality but also act as bioactive molecules that prevent metabolic disorders by modulating the gut-immune axis (59).

### 4.5 Factors influencing biomolecule production

A complex interaction of microbial, chemical, and environmental factors controls the synthesis and accumulation of bioactive metabolites in the fermentation of food and conditions the functional quality of the resulting product. Selection of microbial strains and starter cultures is the main driver of this metabolic diversity. Various LAB and yeasts have unique enzyme repertoires, in particular extracellular proteinases and peptidases, that catalyze the degradation of complex proteins into particular bioactive peptides (65). For example, autochthonous strains are frequently highly proteolytic specific, with higher concentrations of antihypertensive or antioxidant low-molecular-weight peptides than commercial starters (66). Moreover, mixed cultures of traditional fermentation might result in metabolic synergies that allow the cross-feeding, co-metabolism and the complementary activity of the enzymes to produce a wider range of metabolites, including SCFAs and EPS, than mono-culture fermentation (67).

Substrate composition acts as the chemical basis of these transformations as the essential starting materials in the synthesis of biomolecules. The source of proteins whether dairy, plant or marine-based defines the boundary of the variety of bioactive sequences that can be released; e.g., bovine milk protein is abundant in encrypted sequences, which, when hydrolyzed, can release potent ACE-inhibitory peptides (68). In addition to proteins, the carbohydrate profile and the availability of phytochemicals also have a large impact on the growth kinetics of microorganisms and the generation of organic acids. Plant-based fermentation involves enzyme-induced increase of the bioaccessibility of aglycones, which are essential in preventing metabolic disease due to their antioxidant and anti-inflammatory effects during plant-base derived fermentation (69).

These kinetic regulators of fermentation are environmental factors and the duration of fermentation. Both microbial and endogenous enzymes are modulated by parameters including temperature, pH and oxygen availability. Delayed fermentation at inadequate temperatures which is generally the case with the traditional ripening procedures is usually more supportive of biodiversity and the accretive build-up of bioactive fractions (67). On the other hand, rapid acidification at higher temperatures could promote initial proteolysis; however, it can result in the loss of some sensitive metabolites if it is not carefully regulated. The processing and storage of these biomolecules after fermentation are essential to help retain the concentration and stability of these biomolecules (70). Continued enzyme activity throughout storage, commonly called maturation, may cause further refinement of the metabolite profile, although elements such as temperature variation and thermal interventions may result in the degradation of heat-sensitive peptides or vitamins. Stability of these active molecules is required during storage and subsequent gastrointestinal digestion to exert preventive action against metabolic disorders, e.g., glycemic control or lipid regulation, and this suggests that development of the most effective processing conditions is necessary to guarantee physiological functionality (71).

## 5 Interactions between fermentation-derived biomolecules and gut microbiota

### 5.1 Modulation of gut microbial composition and diversity

The biomolecules produced through fermentation such as organic acids, bioactive peptides, EPS, and phenolic metabolites are effective modulators of the gut ecosystem due to their ability to offer certain substrates which remodel the structure of the microbes (72). Compared to the traditional food, fermented food tend to be more bioaccessible or very uniquely structured as a result of microbial biotransformation, and thus can have prebiotic-like selection pressures on commensal populations (3). One major biomolecular route is the enhanced cross-feeding networks, in which primary fermenters degrade complex fermentation-derived polysaccharide or EPS into simpler intermediates, e.g., lactate or succinate, which are then used by secondary fermenters. This symbiotic relationship encourages the growth of saccharolytic niches and increases the generation SCFA or butyrate and propionate, which are necessary in keeping gut homeostasis and host metabolic signaling (73).

The ecological impacts of such biomolecules intake are commonly associated with a major change in the diversity and structure of the microbial communities. Fermented plant polysaccharides and organic-acid-rich beverages are capable of restoring or enhancing alpha diversity that is often depleted in metabolic disorders (74). For example, fermented sea buckthorn juice and *Momordica charantia* polysaccharides enhance community richness and also alter beta diversity to a profile that is linked to better metabolic health as depicted in *in vivo*, C57BL/6 mice model (3). Although the *Firmicutes*/*Bacteroidetes* ratio remains a standard parameter, recently the concentration of particular taxonomic enhancements is more promising to represent functional changes. The most prevalent taxa that are enriched by fermentation-derived metabolites are *Bifidobacterium*, *Lactobacillus*, and *Akkermansia muciniphila*. Consumption of fermentable oligo- and polysaccharides is commonly associated with the growth of *Akkermansia* blooms, which can be mucin-associated degraders and can take advantage of the modified luminal environment (75).

These shifts in composition also have direct implications for the prevention of metabolic diseases like obesity, T2D and non-alcoholic fatty liver disease (NAFLD). An increase in the level of SCFA through the enrichment of beneficial fermenters and subsequent increase in the level of beneficial fermenters is linked to enhanced gut barrier integrity, diminished systemic inflammation, and enhanced glycemic control (76). In high-fat diet models, responder phenotypes (those whose microbiota can effectively ferment the used biomolecules) show less adiposity and elevated insulin sensitivity than non-responders, and this illustrates the significance of individual host-microbiota interactions (72). Moreover, fermentation of polyphenols by microbes transforms the polyphenols into phenolic acids with low molecular weight that inhibit the proliferation of obesogenic taxa and enhances the growth of anti-inflammatory commensals. All these interactions prove that the biomolecules obtained through fermentation are not just adjuncts to the diet but agent of gut microorganisms reprogramming against the physiological causes of the metabolic dysfunction (76).

### 5.2 Microbial metabolic interactions and cross-feeding mechanisms

Investigating the health-promoting functionality of fermented foods presents an increasing number of complex microbial metabolic interactions in the gut, wherein fermentation products form pivotal nodes in vast cross-feeding interactions. These reactions are known as trophic chains, and they involve the transformation of primary fermentation products into secondary metabolites having strong physiological activities beyond nutrient provision (77). The lactate-to-butyrate conversion represents one of the most critical pathways, which helps prevent the harmful build-up of lactic acid that is typically linked with metabolic acidosis and intestinal malfunction in dysbiotic conditions and, conversely, supplies the gut tissue with butyrate. Butyrate is an important energy source for colonocytes, a potent controller of immune homeostasis and the integrity of the intestinal barriers (78). In this symbiotic interaction, *Bifidobacterium*-type primary fermenters digest dietary fibers or EPS within the food matrix creating lactate and acetate as metabolic byproducts. These intermediates are then captured and used by special butyrogenic bacteria, such as Eubacterium hallii and Anaerostipes caccae, which have the special enzymatic apparatus to use lactate and acetate through the butyryl-CoA:acetate CoA-transferase pathway to produce butyrate (79).

In addition to lactate, acetate and succinate act as central exchange metabolites that stabilize the gut ecosystem in a complex pattern of syntrophic growth. Acetate cross-feeding has proven to be a ubiquitous motif in the microbiome; it is the source of the necessary acetyl units needed to sustain chain lengthening operons in secondary fermenters to support the formation of longer-chain fatty acids (80). Microbial intermediates in the TCA cycle, such as succinate, may be released by primary degraders (*Bacteroides*) and then consumed by certain taxa to generate propionate or butyrate, preventing build-up of the intermediate, which has proven to generate pro-inflammatory signals in some cases. Bidirectional culturing experiments showed that these fluxes are necessary in sustaining community diversity and the production of beneficial metabolites which have the potential to support beneficial taxa (81). Moreover, EPS which are formed upon fermentation of food e.g., in yogurt, kefir or sourdough become inducers of these cascades. It is notable that the structural complexity of such EPS molecules necessitates the presence of special primary degraders to enable the enzymatic breakdown of the larger molecules into simpler sugars and organic acids, which supply vitality to the broader metabolic network and enables the survival of non-degrading commensals (82).

These metabolic networks have extensive implications on the general health of the population, especially in the prevention and treatment of metabolic disorders like obesity and diabetes type 2. The carbon flow highly facilitated by fermented food through butyrate and other SCFA allows host-microbiota crosstalk, enhancing insulin sensitivity, stimulating the release of incretin hormones (GLP-1), and decreasing systemic low-grade inflammation (83). Improved glycemic control and diminished adiposity in human cohorts have been systematically linked to enrichment of the taxa including *Blautia wexlerae* that are involved in such advantageous metabolic profiles and trophic chains. Functional fermented foods contribute to the formation of a stable microbial community that ensures resistance to the metabolic imbalances found in modern lifestyle-related illnesses by increasing fecal ecosystems that are robust and diverse in the cross-feeding environment. Metabolic Synergy among fermented foods are not just a source of probiotics, but an intricate bioactive delivery system to coordinate a healthy intestinal ecosystem using bioactive precursors (84).

### 5.3 Effects on intestinal barrier integrity and gut permeability

The integrity of intestinal barriers forms a foundation of metabolic health as it represents a vital point of contact between the host and a web of different microbes inhabiting the gut. The biomolecules produced by fermentation are central to the enhancement of this barrier by complex biomolecular pathways that differentiate the structural and functional characteristics of the intestinal mucosa. Among these, SCFAs, including butyrate at the forefront, stand out as key mediators of the barrier enhancement. Butyrate serves as a major source of energy for colonocytes and directly increases the expression and localization of essential tight junction genes, such as, zonula occludens-1 (ZO-1), occludin and different claudin isoforms (85). These proteins establish a selective barrier between neighboring epithelial cells that control the paracellular permeability and ensure high levels of transepithelial electrical resistance (TEER) blocking the passive diffusion of damaging luminal contents. At the mechanistic level, interaction of SCFAs with G protein-linked receptors e.g., GPR41 and GPR43, induces critical downstream signaling pathways. These comprise the activation of hypoxia-inducible factor-1a (HIF-1a) and interleukin-22 (IL-22) secretion which play vital roles in mucosal homeostasis, epithelial repair and the overall maintenance of barrier function (86).

In addition to SCFAs, the protective effects of bioactive peptides produced during the fermentation of protein-rich matrices are quite significant. For instance, peptides produced by fermentation of dairy, egg-milk, or soybean products help to repair injured epithelium via regulation of complex kinase-related signaling pathways. These are AKT/PI3K and MAPK-NF-κB signaling cascades, which regulate cell survival and inflammatory actions (87). In particular, these peptides suppress the binding of myosin light chain kinase (MLCK), which is the major contributor to tight junction disassembly in inflammatory or in high-fat dietary conditions. Moreover, the synergy of complex fermentation metabolomes, including lactate, indole derivatives, and several amino acid metabolites exhibit provides a high level of protective ability in comparison with single compounds. These signals combine with each other to maintain epithelial cell wall integrity amidst cytokine destabilization, which underscores the significance of entire food fermentation communities (88).

The physiologic importance of a healthy intestinal barrier is best seen in the inhibition of metabolic endotoxemia. This condition is associated with systemic translocation of microbial LPS as a primary source of chronic low-grade inflammation that forms the basis of insulin resistance, obesity, and T2D mellitus. Fermentation-produced molecules effectively reduce permeation of these pro-inflammatory bacterial components through the tight junction complex by strengthening the tight junction complex and enhancing the production of mucus (89). The interventions using fermented products may correct metabolic injury induced by the high-fat diet in animal model, which showed better weight control and level of glycemic regulation (90). Therefore, therapeutic application of functional fermented foods in the population health nutrition creates a powerful tool in preventing metabolic diseases through stabilizing intestinal barrier and adjusting the gut-microbiota-host interface (87).

### 5.4 Immune modulation and inflammatory signaling pathways

The interaction of intestinal fermentation-produced biomolecules interaction with the gut microbiota is an essential axis of regulation of host immune response and systemic inflammatory tone in terms of metabolic disease. Key contenders of this communication are SCFAs, bioactive peptides and microbial EPS, all of which contribute to the recalibration of inflammatory signal pathways (91). The SCFAs, especially butyrate and propionate are the powerful signaling molecules linking microbial metabolic activity to host immune regulation. These metabolites are found to inhibit the classical inflammatory pathways, namely, NF-κB and mitogen-activated protein kinase (MAPK) pathways that are often hyperactivated by obesity and associated metabolic conditions (92). Change in SCFAs profiles caused by certain fermented products, e.g., Limosilactobacillus fermentum, correlates with inhibition of p38/MAPK signaling which, in turn, helps to suppress inflammation and oxidative stress at the tissue level (93).

Alongwith SCFAs, the bioactive peptides produced during fermentation have a unique role in regulating innate and adaptive immunity through balancing microbial composition in the gut. Such peptides, together with probiotic-derived postbiotics, are able to shape the inflammatory environment of the host by regulating the functions of intestinal and systemic immune signaling nodes. These bioactives also have the potential to reduce pro-inflammatory signals and even engage an anti-inflammatory milieu engagement through their binding to pattern recognition receptors (TLRs) (94). Moreover, microbial EPS that are generated during the fermentation of foods such as yogurt and traditional fermented vegetables behave like prebiotic-like substrates that promote the enrichment of beneficial, anti-inflammatory taxa such as *Akkermansia*. This microbial remodeling is closely correlated with a reduction of the systemic inflammatory biomarkers, such as C-reactive protein (CRP), and a reduction in the metabolic burden of low-grade systemic inflammation (95).

**FIGURE 2:**
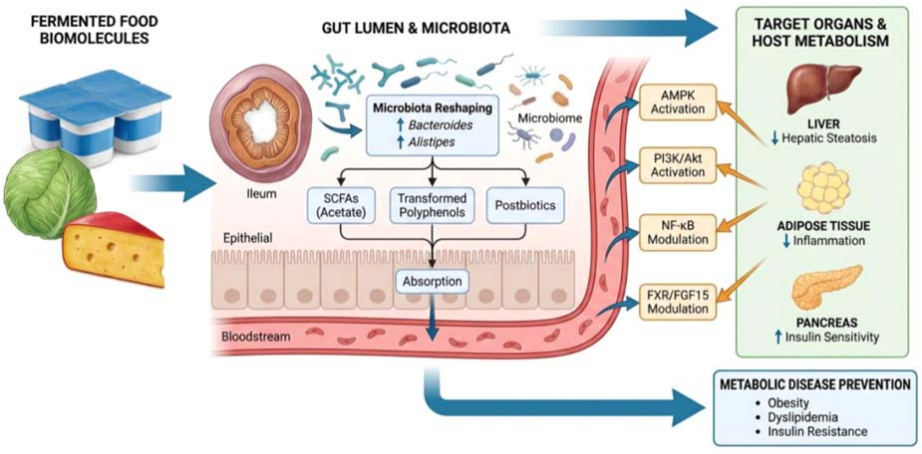
Mechanistic link between fermented food biomolecules, gut microbiota and host metabolism.

Although the general suppression of MAPK and NF-κB signaling is well reported at the pathway level, the precise cellular mechanisms under the regulation of regulatory T cells (Tregs), polarization of macrophages, and maturation of dendritic cells in models of metabolic diseases are currently under investigation. The fermentation-derived metabolites help to decrease the expression of pro-inflammatory cytokines like tumor necrosis factor-alpha (TNF-a) and interleukin-6 (IL-6), which are key mediators of the pathogenesis of insulin resistance and metabolic endotoxemia (96). Through the strengthening of the intestinal barrier and the suppression of an over-activated immune response based on these various biomolecular mechanisms, functional fermented foods represent a provisional nutritional therapy in the prevention and treatment of metabolic diseases (97). Mechanistic link between fermented food biomolecules, gut microbiota and host metabolism shown in Figure 2.

### 5.5 Microbial metabolites as mediators of host metabolic responses

Functional fermented foods have health-promoting effects in the population nutrition o and these effects are mainly mediated by the number of microbial metabolites produced in the fermentation process or as a result of interactions between the fermented food and the gut microbiota. These metabolites, especially the SCFAs, secondary bile acids, and indole derivatives, make them powerful signaling molecules coordinating host metabolic processes through particular G protein- coupled receptors (GPCRs) and intracellular mechanisms (98). SCFAs such as acetate, propionate and butyrate are the primary ligands of the FFAR2 (GPR43) and FFAR3 (GPR41) receptors that are highly expressed in enteroendocrine L-Cells. SCFAs stimulate the secretion of important incretin hormones, including glucagon-like peptide-1 (GLP-1) and peptide YY (PYY), upon stimulation of these receptors, which are necessary for supporting glucose-stimulated insulin secretion, inhibition of appetite, and maintenance of postprandial glycemic fluctuation (99). The study showed that mice with either FFAR2 or FFAR3 deficiency have reduced levels of GLP-1 content and impaired glucose toleration, indicating the essentiality of metabolite-sensing GPCR in glucose homeostasis. In addition to incretin axis, microbial products mediate the modulation of peripheral energy metabolism and insulin sensitivity by stimulating adenosine monophosphate-activated protein kinase (AMPK). SCFA accessing the liver and skeletal muscle have the capacity to stimulate AMPK-linked pathways that encourage mitochondrial fatty acid oxidation and restrain lipogenesis thus averting the onset of NAFLD and hepatic steatosis (100). Moreover, the secondary bile acids, which are the result of the microbial processing of the primary bile acids, transmit via the TGR5 (GPBAR1) receptor. TGR5 stimulates L-cells to further boost GLP-1 secretion and synchronize the release of pancreatic hormones and when TGR5 is found on brown adipose tissue and muscle it increases energy expenditure, providing a multi-faceted solution to weight management and obesity prevention (96).

**FIGURE 3:**
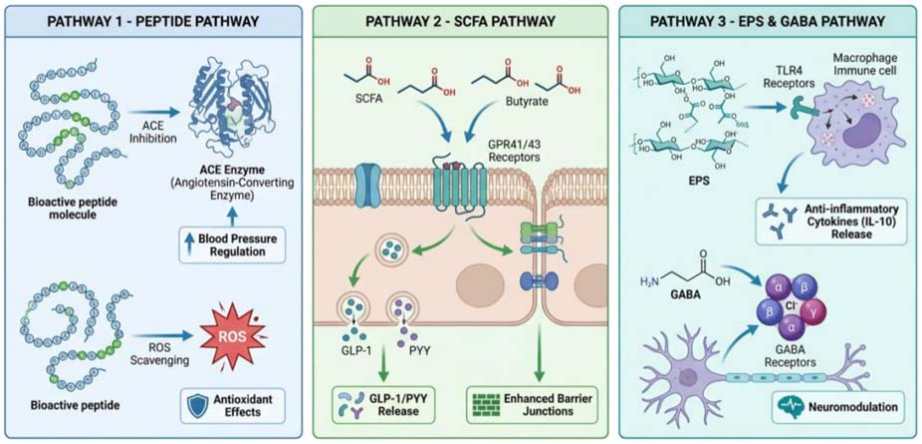
The molecular mechanism of fermented derived biomolecules.

Another important group of mediators, produced after tryptophan conversion by the microbial metabolism, are indole derivatives, which regulate enteroendocrine sensing and gut barrier activity. These metabolites have the capacity to stimulate the aryl hydrocarbon receptor (AhR), and other signaling to induce GLP-1 production and intestinal integrity, which is crucial in preventing the systemic low-grade inflammation usually seen in the context of metabolic syndrome (101). Although direct signaling of fermented-food-derived bioactive peptides into SCFA-specific GPCRs is still a subject of research, fermented foods prove most effective as a delivery system for these metabolic precursors and their microbial communities. Functional fermented foods together enhance insulin sensitivity, alleviate systemic inflammation and improve satiety by modulating the luminal metabolite profile, which helps in the prevention of obesity, T2D and metabolic syndrome (102). The molecular mechanism of fermented derived biomolecules illustrated in Figure 3.

## 6 Biomolecular mechanisms in metabolic disease prevention

### 6.1 Regulation of glucose metabolism and insulin sensitivity

Complex, multi-layered biomolecular pathways mediate the regulation of glucose metabolism, and functional fermented foods improve insulin sensitivity through carboxylic acid (SCFAs) and bioactive peptide fermentation-derived metabolites. The activation of AMPK pathway is one of the major mechanisms. The fermented soybean extracts (e.g., AEH) and individual bioactive peptides (e.g., VY and SFLLR) can substantially induce AMPK phosphorylation in insulin-responsive tissues (e.g., skeletal muscle and the liver) (103). AMPK is a metabolic master switch that stimulates glucose clearance by inhibiting hepatic gluconeogenic enzymes, namely, phosphoenolpyruvate carboxykinase (PEPCK), and glucose-6-phosphatase (G6PC), and stimulates glucose uptake in skeletal tissues (104).

Moreover, translocation of glucose transporter 4 (GLUT4) to the plasma membrane is triggered by the consumption of fermented products, such as fermented teas and berry juices. This is stimulated by the dual activation of phosphatidylinositol 3-kinase (PI3K)/Akt and AMPK pathways (105). For instance, fermented lowbush blueberry juice has been shown to increase the glucose absorption by myotubes and adipocytes through the induction of GLUT4 traffic, whereby it lowers postprandial glycemic excursions in db/db mice model. This enhanced membrane recruitment of GLUT4 is a key mechanism in overcoming insulin-resistant states and restoring homeostatic glucose handling in models of metabolic diseases (106).

In addition to direct cellular signaling, fermentation obtained SCFAs, specifically butyrate, are also critical in the regulation of glucose in the body via the gut-endocrine axis. Butyrate is a G-protein coupled receptor agonist that interacts with free fatty acid receptor 2 (FFAR2 or GPR43) and GPR41 on intestinal L-cells. Stimulation of these receptors leads to the release of incretin hormones with glucagon-like peptide-1 (GLP-1) being the most prominent of them and contributing to the increase in glucose-dependent insulin secretion by pancreatic beta cells and improved overall insulin sensitivity (107). The anti-diabetic activity of some probiotic strains such a as *Leuconostoc mesenteroides* is significantly reliant on this SCFA-receptor interaction in type 1 diabetic mice, as the metabolic activity is significantly reduced when GPR43 is knocked down (108).

Moreover, enrichment of the SCFA-generating bacteria through the intake of fermented foods causes a self-stimulating mechanism that sustains both high levels of systemic GLP-1 and low levels of metabolic endotoxemia, which further stabilizes the glucose homeostasis (109). Fermented polyphenols and peptides also act by alpha-glucosidase-inhibition, which results in lower uptake of carbohydrates, but recent studies have shown intracellular signaling and incretin-mediated pathways as the prevailing controlling factors in enhancing insulin sensitivity through functional fermented foods.

### 6.2 Effects on lipid metabolism, cholesterol balance, and adiposity

Functional fermented foods have a potent effect on lipid metabolism and adiposity via the multi-faceted network of biomolecular regulation. One major pathway is the downregulation of transcriptional programs of adipogenesis and lipogenesis. Biomolecules produced as a result of fermentation, especially bioactive peptides and certain probiotic metabolites suppress the expression of peroxisome proliferator-activated receptor gamma (PPAR-γ) and CCAAT/enhancer-binding protein alpha (C/EBPα) (110). These master regulators play a vital role in adipocyte differentiation; their inhibition along with the down-regulation of downstream effector proteins including fatty acid synthase (FAS) and acetyl-CoA carboxylase (ACC) profoundly reduces fatty tissue gain in the adipose tissue (111). On the other hand, some of the fermented products trigger the transition to lipid catabolism through the up-regulation of PPAR-α and AMPK pathway. AMPK activation enhances hepatic and muscular β-oxidation of fatty acids, but decreases *de novo* lipogenesis, which is an efficient way to reduce triglyceride and low-density lipoprotein (LDL) levels in the body (110).

Bile acid metabolism also contributes to cholesterol homeostasis and this process is heavily dependent on fermented microbial metabolism. Fermented foods contain probiotic strains that have bile salt hydrolase (BSH) activity, which promotes the breakdown of bile salts in the small intestine. Deconjugated bile acids are not reabsorbed as effectively, and fecal excretion becomes more frequent as a result (112). This cholesterol-reducing effect is enhanced by upregulating hepatic cholesterol 7α-hydroxylase (CYP7A1), the rate-limiting enzyme in bile acid synthesis to transform systemic cholesterol to bile acids. Although the direct implication of a farnesoid X receptor/FXR or fibroblast growth factor (FGF19/21) signaling is also observed in gut fermentation, overall alteration of the bile acid pool is an essential bridge linking gut fermentation to lipid profiles in the body (113).

Also, the fermentation enhancement of SCFA, such as butyrate and propionate, through changes in gut microbiota are essential to cholesterol homeostatic processes. The amount of fecal butyrate has been found to have a negative relationship with total cholesterol and LDL-C, which indicates that SCFAs could contribute toward the inhibition of hepatic cholesterol production or improved clearance (114). Moreover, fermented food models promote a systemic condition favoring less adipose and better metabolic health via upregulation of lipid-clearing enzymes such as lipoprotein lipase (LPL) and carnitine palmitoyltransferase 1 (CPT1) (110). Taken together, the combined effect of these mechanisms (gene silencing in adipocytes to change in the bile acid flux) make functional fermented foods a powerful dietary intervention in the prevention of hyperlipidemia and metabolic disorders caused by obesity.

### 6.3 Anti-inflammatory and antioxidant signaling pathways

Functional fermented foods have strong protective effects on metabolic diseases such as obesity, T2D, and NAFLD, which are mainly mediated by the regulation of anti-inflammatory and antioxidant signal pathways. One of the core pathways is the suppression of the NF-κB pathway, which is the primary controller of the inflammatory reaction. Fermentation of proteins into bioactive compounds, including the selective phenolic metabolites and probiotic-generated factors, inhibits the nuclear translocation and phosphorylation of the NF-κB p65 subunit (115). This inhibition causes a great decrease in the transcription and systemic release of pro-inflammatory cytokines, especially TNF-α and IL-6, which play a major role in insulin resistance and chronic low grade inflammation in metabolic syndrome (116). In addition to these anti-inflammatory effects, these are supported by activation of nuclear factor erythroid 2-related factor 2 (Nrf2) and heme oxygenase-1 (HO-1) antioxidant axis. Fermented extracts, such as those of *Bacillus subtilis* or of LAB, increase the expression of Nrf2 which in turn leads to the expression of vital antioxidant enzymes such as superoxide dismutase (SOD), glutathione (GSH), and catalase (CAT). These fermented products do so by increasing the antioxidant capacity of cells to counteract oxidative stress, which takes the form of decreased malondialdehyde (MDA) and reactive oxygen species (ROS), and hence protects the hepatocyte and pancreatic beta cells against lipotoxic and glucotoxic damage (117). In addition, functional fermented foods regulate MAPK and Toll-like receptor 4 (TLR4) signaling pathways. These diets suppress TLR4 sensing of gut-derived endotoxin (LPS) which is often exaggerated in obesity because of elevated intestinal permeability. Fermented bioactives also suppress the downstream MAPK cascades, especially c-Jun N-terminal kinase (JNK) pathway, thus inhibiting NF-κB activation besides while enhancing insulin receptor signaling, which subsequently ameliorates hepatic steatosis and hepatic glucose intolerance systemically (14).

The coordination of these pathways is, to a largest extent, provided by the variety of biomolecules produced via fermentation. Phenolic compounds which are converted to more bioavailable and bioactive forms by microbial enzymes represent direct activators of Nrf2 and inhibitors of NF-κB (118). Bioactive peptides, which are liberated in food when proteins are broken down by microbes, also play a role in these effects by regulating redox sensitive kinases and inflammatory gene expression. Also, EPS and other postbiotics have a direct or indirect influence on these host responses through remodeling the gut microbiota, which decreases endotoxin load in the system and creates a more anti-inflammatory luminal environment (119). These interlocked biomolecular processes imply the effectiveness of functional fermented food as a viable policy tool that can be used in primary prevention and management of metabolic illness in the general population. The metabolic benefits of fermented foods summarized in Table 2.

**TABLE 2:** Metabolic benefits of fermented foods.

Fermented food	Primary bioactive components	Impact on insulin sensitivity/ glucose	Impact on lipid profile/ cholesterol	Impact on weight/ adiposity	Anti-inflammatory effects	References
Yogurt	Branched chain hydroxy acids, probiotics (*Lactobacillus*, *Bifidobacterium*)	Preserves glucose homeostasis; prevents hepatic insulin resistance.	Reduces LDL-C and improves serum/liver lipids.	Prevents liver steatosis; promotes fat browning and energy expenditure.	Modulates gut microbiota and reduces metabolic inflammation.	(120)
Kefir	Probiotic consortia (bacteria and yeasts), kefiran (exopolysaccharide)	Significant reduction in fasting insulin and HOMA-IR; improves glycemic control in T2DM.	Mixed results; some studies show no consistent effect on TC or TG in humans.	Generally no significant effect on total body weight in pooled RCTs.	Reduces pro-inflammatory cytokines (IL-1β) and increases IL-10.	(121)
Kimchi	*L. plantarum* K50, SCFAs, capsaicin (from chili)	Potential glycemic benefits via SCFA production.	Reduces serum triglycerides and increases HDL-C.	Decreases total body and regional adipose tissue weight; reduces liver fat.	Downregulates pro-inflammatory markers (TNFα, IL-1β).	(122)
Sauerkraut	LAB, lactic acid, GABA, phenolic compounds	Fasting glucose improvements (primarily in animal/botanical fermented food trials).	Lowered LDL-C and triglycerides (animal models); downregulates lipogenesis genes.	Reductions in body weight and white fat pad weights in rodent models.	Protects intestinal barrier; reduces CRP in botanical fermented food trials.	(123)
Kombucha	Acetic acid bacteria, yeasts, tea polyphenols, organic acids	Acute postprandial reduction in glycemic and insulin indices.	Reduced total cholesterol and TG in diabetic rats; mixed human evidence.	–	Inhibits inflammation linked to insulin resistance via gut barrier protection.	(123)
Tempeh	Fermented soy proteins, bioactive peptides, LAB-enriched soy	Improved glucose and insulin levels in diabetic rodent models.	Decreased LDL-C and improved liver lipids in hypercholesterolemic models.	–	Reduced metabolic inflammation when combined with antioxidants.	(124)
Natto	*Bacillus subtilis* natto, nattokinase, viscous fiber	Improved composite insulin sensitivity index (CISI) and acute postprandial glucose.	Significant reduction in total cholesterol and LDL-C in overweight subjects.	–	Reduced oxidative stress markers; potential anti-atherogenic effects.	(125)

### 6.4 Gut-liver axis and reduction of metabolic endotoxemia

The gut-liver axis represents a bidirectional communication system that plays a pivotal role in the pathogenesis of metabolic diseases, including NAFLD, obesity, and T2D. A hallmark of these conditions is metabolic endotoxemia, characterized by a chronic, low-grade elevation of LPS derived from the cell walls of Gram-negative gut bacteria. Functional fermented foods have emerged as potent modulators of this axis, primarily by reinforcing the intestinal epithelial barrier and preventing the translocation of endotoxins into the portal circulation (126).

Fermented food exert protective effect through the enhancement of intestinal barrier integrity. Consumption of these foods, particularly those enriched with specific probiotics like *Lactobacillus plantarum*, has been shown to upregulate the expression of essential tight-junction proteins, such as zonula occludens-1 (ZO-1), occludin, and claudins in mice (127). By restoring the physical and functional architecture of the gut epithelium, fermented foods significantly reduce intestinal permeability, often referred to as leaky gut. This structural reinforcement is further supported by fermentation-derived metabolites like SCFAs such as butyrate and propionate, which provide energy for colonocytes and stimulate the production of mucins, thereby strengthening the biochemical barrier (128).

**FIGURE 4:**
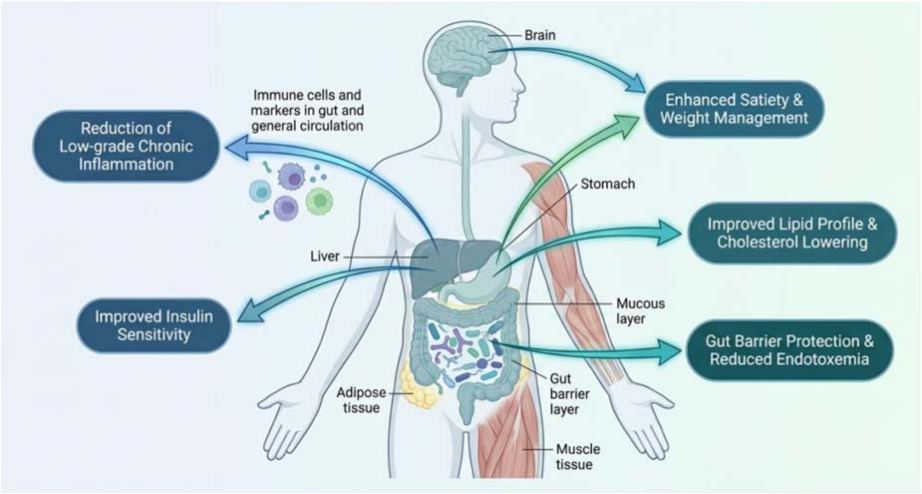
Implication of fermented food for metabolic disease prevention.

The subsequent reduction in the translocation of LPS from the intestinal lumen into the portal vein is a critical step in mitigating metabolic endotoxemia. Lower levels of portal LPS diminish the chronic activation of hepatic TLR4 signaling. In the liver, the inhibition of the LPS/TLR4/NF-κB pathway leads to a marked decrease in the production of pro-inflammatory cytokines, such as tumor necrosis factor-alpha (TNF-α) and IL-6, and suppresses the activation of the NLRP3 inflammasome (129). These anti-inflammatory effects, coupled with improvements in microbial lipid metabolism, result in reduced hepatic steatosis and the prevention of progression from simple fatty liver to more severe forms of metabolic dysfunction (130). By restoring gut-liver homeostasis and reducing the systemic inflammatory tone, functional fermented foods provide a promising nutritional strategy for the prevention and management of NAFLD and associated metabolic disorders. The implication of fermented food for metabolic disease prevention shown in Figure 4.

### 6.5 Integrated microbiota-host metabolic network interactions

The systemic metabolic landscape of the host is redefined by a cascade of biomolecular interactions triggered by the ingestion of functional fermented foods by maintaining a multilayered hierarchy. This is now being comprehended in multi-omics frameworks-integrated metagenomics, metabolomics and host transcriptomics, in which fermentation-derived metabolites are found to interact not in isolation but are instead as a part of massive signaling nodes within a large microbiota-host metabolic network (131). These metabolites, such as SCFAs, secondary bile acids and amino acid derivatives, which ultimately communicate the gut lumen-origin signal to distant metabolic tissues (liver and adipose tissue), ensuring that energy homeostasis and inflammatory tone of the host are aligned (2).

One of the main pathway is the gut-liver axis, where changes in microbial metabolome resulting from fermentation directly reestablish a new gene expression in liver. For instance, co-fermented whole grains such as quinoa and black barley change the fecal pool of oxalic acid and dihydroxycholesterol derivatives which modulate the hepatic transcriptomic programs of glucose and lipid metabolism, including *Gck* and *Cidea*. Additionally, microbiota-produced SCFAs are also key ligands for both the aryl hydrocarbon receptor (AhR) and G-protein coupled receptors of the intestine and liver, which regulate expression of host genes that control systemic metabolite concentrations and hepatic steatosis (132). This bidirectional communication plays a critical role in the prevention of metabolic diseases since it enables the host to adjust its metabolic flux corresponding to the particular nutrient and microbial composition offered by fermented matrices (133).

These integrated systems exert effects on adipose tissue alongwith the liver, where microbial signals respond to adipocyte differentiation and the lipid storage capacity. Human cohort clustering Multi-omics of the fecal metabolome has identified certain associations between fecal-metabolite cluster groups (e.g., indoles and individual SCFAs) and cardiometabolic risk systemic lipid markers such as sphingomyelins and acylcarnitines (134). Fermented drinks and soybean-based functional drinks have been indicated to enhance serum level of anti-inflammatory lipid mediators and bioenergetic precursors, which organize a systemic response characterized by increase oxidative capacity as well as reduced low-grade inflammation (135). Fermented foods replace the obesity and metabolic syndrome dysregulation by restoring a sustained provision of network-stabilizing metabolites through restructuring the gut ecosystem to support the beneficial taxa such as *Akkermansia* and *Lactobacillus*. The combination of these integrated interactions shows that fermented foods act as precision modulators of the metabolic architecture of the host and provide a sound approach to the population nutrition (2).

## 7 Evidence linking fermentation-derived biomolecules to metabolic health outcomes

The evidence linking functional fermented foods to improved metabolic health outcomes spans a hierarchical spectrum from fundamental molecular investigations to large-scale population studies. Mechanistic *in vitro* analysis have defined a wide range of fermentation biomolecules which directly connect with host metabolic pathways. Organic acids, including acetic acid secreted in the milks fermented by *Bifidobacterium*, have been reported to promote glucose absorption and adenosine AMPK signaling in peripheral tissues (136). One notable recent finding suggesting the existence of cell-autonomous mediators is the identification of branched-chain hydroxy acids (BCHA), highly increased in yogurt due to microbial proteolysis, which have a direct beneficial effect on insulin in hepatocytes and myocytes. Moreover, fermentation of phytochemicals leads to their microbial refinement and formation of potent bioactive compounds, for example, fermented tea polyphenols such as theaflavins and theasinensin A have been shown to inhibit intestinal fat absorption as well as control postprandial glucose responses (137). Similarly, the antimicrobial kombucha extracts of cascara have an inhibitory potential toward α-amylase and lipase, implying mechanistic inhibition leading to reduced carbohydrate and lipid digestions (138).

There is strong evidence on the beneficial effects of fermented food in animal models, predominantly rodent models fed high-fat diets (HFD), supporting systemic effects on fermented food consumption. Obese mice treated with yogurt have been reported to maintain whole-body glucose homeostasis and prevent the emergence of hepatic insulin resistance and steatosis, both strongly mediated by tissue-specific levels of BCHA and intestinal microbiota remodeling (139). Plant-based fermentation is also effective; ginseng fermented using *Monascus* has been identified to mitigate liver steatosis and hyperlipidemia by upregulating hepatic p-AMPK as well as supporting bile acid metabolism (140). In other studies employing the use of *Lactobacillus*-fermented cabbage-apple juice in HFD rats, significant decrease in body weight, fat pad mass, serum triglycerides, and fasting glucose were observed due to the down-regulation of hepatic lipogenic genes. These models consistently demonstrate that fermented products have better protection against metabolic dysfunction than the non-fermented base food components regardless of variation caused by the microbial strain and food matrix (141).

Prospective cohort studies and clinical trials repeatedly show that yogurt consumption can reduce the occurrence of T2D (139). Yogurt and kefir randomized controlled trials (RCTs) have shown a significant effect on fasting glucose, insulin sensitivity, and systemic inflammatory factors, but less effect were observed on weight reduction and body mass index (BMI),. Although positive changes in LDL-cholesterol and blood pressure are observed in some of the studies, these findings are not consistently observed, frequently being characterized by variations in intervention duration, dosage, and pre-intervention metabolic condition of participants (2).

Epidemiological data provide a longitudinal analytical account of the role of fermented foods in the global diet. A dose–response meta-analysis of 22 prospective human cohort studies (579,832 participants; 43,118 T2D cases) showed that total dairy consumption was modestly inversely associated with T2D risk (RR: 0.97 per 200 g daily intake). Notably, yogurt intake (80 g/day) was associated with a 14% lower T2D risk (RR: 0.86; 95% CI: 0.83–0.90), suggesting fermented dairy may aid T2D prevention, with consideration of study heterogeneity (142). Comparable, relationships have been observed between fermented soy foods such as natto and miso with Asian populations, where consumption has been associated with lower CVD mortality and improved metabolic syndrome phenotype (143). Such dietary habits demonstrate the holistic advantage of fermented foods as delivery systems not only of probiotics but also of specialized metabolites created during the food fermentation. Although these findings are promising, critical analysis presents a number of research inconsistencies and limitations. The main advantage of the field is its mechanistic depth, in which the multi-omics study in animal models has been effective in discovering individual (metabolites) such as BCHA, which connect the food matrix to physiological reactions of host organisms (139).

However, there are a number of factors which inhibit the translation to human health. The small size of most RCTs, as well as the enormous variability of fermented foods types, microbial communities and fermentation time periods, renders it challenging to develop uniform clinical guidelines (144). In addition to this, translational gap exists between robust metabolic improvements observed in rodents and comparatively few study in human trials. These discrepancies should be addressed in future studies by applying standardized fermentation product, clearly defined clinical outcomes and longitudinal designs that consider variability of individuals in the gut microbiota, which ultimately determinant of the host capacity to process and benefit of fermentation derived biomolecules (2). Table 3 summarize the evidence linking fermented foods to metabolic outcomes.

**TABLE 3:** Evidence linking fermented foods to metabolic outcomes.

Study type	Population/ model	Fermented food intervention	Duration	Key biomolecules/mechanisms identified	Main metabolic findings (significance levels, % risk reduction)	Reference
*In vitro*	Caco-2, HepG2 cells	Dark tea (theabrownins)/postbiotics	24–48 h	Activation of AMPK/PGC1α, PI3K/Akt, and inhibition of NF-κB	∼30% reduction in intracellular lipid accumulation; significant downregulation of inflammatory cytokines (*p* < 0.05)	(19)
Animal	HFD-induced obese mice	Fermented purple potato dough	8–12 weeks	Altered PE-NMe glycerophospholipids; increased *Blautia* and *Ligilactobacillus*	Significant reduction in body weight gain, TC, TG, and LDL-C; improved glucose tolerance (*p* < 0.01)	(145)
Animal	Diet-induced obese mice	Rice kefiran (*L. kefiranofaciens*)	8 weeks	Increased fecal acetate; microbiota reshaping (*Bacteroides*↑, *Alistipes*↑)	Suppressed weight gain; significant reduction in hepatic steatosis and improved insulin sensitivity (*p* < 0.05)	(146)
Clinical trial	Adults with metabolic syndrome	Fermented dairy (yogurt/kefir)	12 weeks	Precision modulation of gut microbiota; production of bioactive peptides	15–20% reduction in C-reactive protein (CRP); significant improvement in HDL/LDL ratio (*p* < 0.05)	(147)
Epidemiological	Large human cohorts (prospective)	Probiotic-rich fermented foods	Multi-year	Interaction between fermentation-derived metabolites and host signaling	∼12% lower risk of developing T2D; associated with lower BMI and waist circumference	(143)

## 8 Optimizing fermentation for enhanced biomolecule production and bioavailability

### 8.1 Selection of starter cultures and microbial consortia

The functional fermented food optimization process depends on the strategic choice of starter cultures and microbial consortia with a particular set of genetic and metabolic characteristics that stimulate *in-situ* generation of health-promoting biomolecules. One of the most important selection criteria for starters, especially in dairy and soy-based systems, is availability of powerful proteolytic systems, such as cell-envelope proteinases (CEPs), intracellular peptidases and peptide transporters (26). These enzymatic machines play a role in the sequential release of bioactive peptides from precursor proteins; an example of these is the CEPs, which trigger the hydrolysis of intact proteins into oligopeptides that are then further cleaved into shorter, bioavailable sequences with known antihypertensive, antioxidant and immunomodulatory effects. Moreover, accumulation of gamma-aminobutyric acid (GABA) a non-protein amino acid that regulates neuroendocrine signaling and blood pressure, requires the selection of strains possessing glutamate decarboxylase (GAD) system (148). EPS biosynthetic operons and glycosyltransferases producing strains are also screened, as they define the amount and complexity of the EPS, thus affecting the techno-functional characteristics of the food and its use as a prebiotic substrate to biosignal through the gut microbiota (149).

Although single, well-characterized strains are easy to work with and predictable in their yield, enabling improved industrial process control, particularly over specific metabolites such as a single peptide or GABA, microbial consortia or co-cultures are more likely to offer enhanced metabolic synergy (150). In these intricate ecologies, as represented by the artisanal kefir or specially multi-strain starters, metabolic products of one organism may be used as the substrate by another-so-called cross-feeding. For instance, a highly proteolytic *Lactobacillus* isolate can release amino acids that can subsequently be transformed by, GAD-positive co-culture isolate into GABA, producing greater total concentration of bioactive molecules than each isolate alone. Furthermore, consortia tend to be more substrate-flexible and resilient, thus amplifying the output of diverse metabiotics in various raw materials. Beyond production, high enzymatic activity of the starters is also selected to enhance the biomolecules bioavailability by digesting antinutritional factors (e.g., phytates, tannins) and enhancing the solubility of micronutrients (151). This optimization can be done operationally through dual strategy, screening the key functional genes (e.g., *gad* for GABA, EPS operons) using molecular screening, and validating the stability and synergetic performance of the consortia using phenotype-based pilot fermentations. Researcher can optimize the delivery of functional biomolecules by using these microbial selections to the precise nutritional matrix to coordinate systemic host metabolic responses via the gut-liver-adipose axis (152).

### 8.2 Substrate optimization and food matrix engineering

Optimization of fermentation substrates and the strategic design of the food matrix are important for maximization of production and systemic bioavailability of health promoting biomolecules. The substrate used directly determines the resultant microbial metabolic output; such as the addition of fermentable prebiotics, like inulin and fructo-oligosaccharides (FOS), provides a predetermined source of carbon that shifts microbial metabolism by producing SCFAs and organic acids, both *in vitro* during fermentation and during the transit through the gut (153). Surprisingly, the food vehicle can profoundly affect the efficacy of these prebiotics; the similar concentrations of the same prebiotics in various dairy matrices can trigger divergent metabolic effects indicating that food matrix components such as fats and proteins can regulate the speed at which the prebiotics are fermented and the resulting production of metabolites in the host (154).

Biomolecule enrichment has special prospects with food matrix engineering [specially the transition between dairy and plant-based (pulse) matrices]. The pulse-based matrices, such as common beans or the chickpeas have a high density of protein reservoir, which can be used as a substrate by the microbial proteolysis process to release bioactive peptides with potential antihypertensive and antioxidant effects (69). In addition, fermentation in these pulse matrices has a dual effect, as it destroys antinutritional components such as phytates and tannins, with increasing the bioavailability of essential minerals and amino acids and facilitating the synthesis of postbiotic compounds (2).

The bioavailability of these compounds is further enhanced through technological interventions. Although modern technology creates microencapsulations to preserve vulnerable metabolites during gastrointestinal processes, while the basic process optimizations include enzymatic pre-treatment, controlled thermal processing and accurate selection of fermentation kinetics. Time and temperature is the most important factor in improving EPS, GABA, and peptides yield in functional food products. Indicatively, the enzymatic structural changes in plant-based yogurts have been explored to maximize the release of bioactive compounds, as well as, increasing sensory stability (155). Finally, prebiotic-substrate synergies combined with predesigned matrix architecture are essential to guarantee that fermentation-produced biomolecules are produced not only in large quantities but also efficiently delivered to the target locations in the host and, therefore, maximize their protective potential in preventing metabolic diseases (156).

### 8.3 Controlled fermentation technologies and innovations

The technology of controlled fermentation has developed greatly, shifting the traditional spontaneous systems to more precise systems that make the most of the bioactive metabolites localization. The concept of precision fermentation requires the utilization of known co-cultures and defined inoculation orders to trigger production of certain compounds such as GABA and bioactive peptides (157). For example, serial co-fermentation of *Lactobacillus plantarum* followed by Acetobacter aceti on rice wine lees has been demonstrated to be capable of producing substantial amounts of GABA, whilst preserving the sensory properties of functional vinegar. Also, in milk, the presence of *Bacillus subtilis* and *Lactiplantibacillus plantarum* increases the concentration of GABA and other functional biomolecules such as gamma-polyglutamic acid (γ-PGA) and antihypertensive peptides (158). These precision measures are based on pre-treatment of the substrate, including incorporation of precursors such as monosodium glutamate (MSG), and careful control of environmental conditions, including pH and temperature, required to generate repeatable and high-potency metabolic profiles (159).

In order to overcome stability and bioavailability issues, recent delivery mechanisms, including microencapsulation, are being incorporated into the production of functional fermented foods. A large number of fermentation-derived bioactives, especially small peptides and aglycones, are very vulnerable to enzyme degradation by the gut microflora or degradation during industrial processing and storage (160). Food-grade polymers are used to encapsulate these molecules to protect them against adverse gastric conditions, after which they are released in control manner in the small intestine to induce systemic action. Moreover, spray-drying and matrix tuning developments make it possible to produce stable concentrated powder forms of fermented extracts that retain functional potency from production to consumption such as ACE-inhibitory or antioxidant activity (161). This technological layer is needed to enable the conversion of labile fermentation products into dependable nutraceutical tools for metabolic health management.

Non-thermal processing technologies such as High-Pressure Processing (HPP), ultrasound-assisted extraction and other technologies are further refined to enhance the content and bioavailability of fermented bio-molecules. The technologies are employed to enhance the mass transfer of intracellular bioactives into the food matrix without the thermal damage as experienced in the conventional pasteurization (162). The ultrasound-aided fermentation may disrupt the cell walls of the microbes to release desirable enzymes and peptides, whereas HPP is progressively used after fermentation to inactivate fermentative microbes without compromising heat-sensitive antioxidants and phenolic metabolites. Together with precision fermentation, the non-thermal techniques can greatly increase the extraction performance and functional role density of the final product (163). A combination of these forms of these technology have significant potential in the field of public health to prevent and treat metabolic ailments such as obesity and T2D through the provision of a steady and bioavailable dosage of functional biomolecules thereby enabling uniform dietary interventions (160).

### 8.4 Strategies to improve stability, delivery, and bioavailability of biomolecules

The therapeutic properties of fermentation-produced biomolecules, including bioactive peptides, bioactive amino acids, and SCFAs, are frequently restricted due to their solubility to environmental stress and adverse environment of the GIT. In order to counter these problems, advanced food engineering measures are aimed at improving the stability and controlled delivery of such compounds. Microencapsulation has been one of the cornerstone methods and is based on the use of food grade biopolymers such as alginate, nanocrystalline starch, and multilayer emulsions to physically protect bioactive agents against gastric acidity and enzyme degradation (164). For instance, nanocrystalline starch-alginic acid gel has shown encapsulation efficiencies of more than 89% and is much more effective in the survival of encapsulated probiotics and retention of small molecules such as GABA during sequential digestion. Multilayer co-encapsulation, like double-emulsions (W1/O/W2), has demonstrated longevity of greater than 70% of GABA as well as the viability of the microbial consortia through GIT (165).

The selection of drying technology is also very important for determining the industrial scalability and molecular integrity of these functional ingredients. Compared to freezing-dried and spray-dried products, comparative analysis of encapsulation efficiencies show different tradeoffs, with freeze-drying often providing higher encapsulation efficiencies (up to 95% encapsulation of specific probiotics and 90% encapsulation of GABA) whereas spray-drying can be used to produce higher quality of powder, which has higher flowability and increased thermal stability (166). The use of EPS or maltodextrin as wall material give the best survival rate of around 92.5% with an optimized spray-drying formulation maintaining intact bioactive payloads after processing and storage (167).

Moreover, synbiotic and composite systems provide dual-layered bioavailability. The combination of prebiotic wall materials, including inulin, fructooligosaccharides (FOS), or microbial EPS, into the delivery matrix can simultaneously provide physical protective and a fermentable substrate that promotes microbial recovery, and the production of metabolites in the colon. The ability of these synbiotic powders to decrease colonic β-glucuronidase activity of animal models suggests that the delivery system can proactively alter the gut functional environment (168). Although microencapsulation has key protective effects, secondary protection also exists through the presence of an encapsulated protein within a food environment, e.g., it has been observed that encapsulated peptides introduced within food environment; for example, encapsulated peptides introduced into lipid-rich foodstuffs such as peanut butter are supported by the food environment to promote intestinal recovery and bioavailability (169). Naturally, a combination of the above technological interventions can be consider as an advanced set of instruments used to ensure delivery of the fermentation-derived biomolecules to the distal gut, thereby providing a maximum effect on host metabolic health and systemic pathways (164). Strategies for optimizing the bioavailability and bioactivity of fermentation-derived molecules summarized in Table 4.

**TABLE 4:** Strategies for optimizing the bioavailability and bioactivity of fermentation-derived molecules.

Optimization strategy	Impact on biomolecule production	Effect on bioavailability/stability	Technological innovation involved	References
Co-culture systems	2-fold to 10-fold increase in target lipopeptides (e.g., Fengycin titer up to 2.4 g/L); complete detoxification of alkylamides in 3 days	Enhanced metabolite diversity and synergistic production of complementary bioactives	Designed multi-strain consortia (e.g., 4BS, 5BS) and sequential fermentation	(170)
Substrate fortification and media optimization	Significant increases in crude protein (up to 14.75 mg/g), total phenols (6.03 mg/g), and free amino acids (up to 64.8%)	Peak release of water-soluble markers during intestinal phase of digestion	Response surface methodology (RSM); mixed-sugar fermentation models	(171)
Encapsulation (nano/micro)	Stabilized production of phenolic-rich fermentates; enhanced retention of antioxidant activity (up to 45%)	133–165 nm carrier sizes; improved resistance to gastric transit and controlled intestinal release	Nanoencapsulation (HyperCelle); bioencapsulation of Andean grain peptides	(172)
Omics-guided starter selection	Targeted production of high-value metabolites like urolithin A; identification of metabolic bottlenecks	Improved survival and colonization of gut-active strains; verified urolithin bioavailability	Metabolomics-guided strain selection; 16S/shotgun metagenomics; LC-MS/MS	(173)
Microfluidics-assisted processing	Enhanced peptide stability and precise formulation of functional food ingredients	Sustained release of bioactive markers; prevention of premature degradation	Microfluidic-based nano-emulsification and particle formation	(174)

## 9 Translation into public health nutrition strategies

### 9.1 Integration into dietary guidelines and preventive nutrition frameworks

The inclusion of fermented foods in national dietary guidelines is a new horizon in the public health nutrition. Although the historical and cultural role of such foods cannot be denied, their explicit classification as a desired food category is not consistent across international nutrition models. However, at present, fermented products are formally accepted in policy food guidelines of only a few countries, such as Canada and India, as well as in regional food patterns, such as the Mediterranean diet (175). The Canada Food Guide considers yogurt and kefir as important sources of dairy, whereas the Indian guidelines promote local fermented foods as idli and curd due to their greater digestibility and better bioavailability of the nutrients (176). Fermented vegetables and dairy are considered core elements of the Mediterranean diet linked to the long-term cardiometabolic health (1). However, in many countries, there is still no specific recognition of fermented foods, and that they are usually classified by their raw material (e.g., dairy, soy), but not based on their distinct microbial and biomolecular fingerprint (177).

The lack of a standardized definition of fermented foods as a global standard is a major obstacle in determining the formal public health recommendations. Policy frameworks frequently fail to make a distinction between the probiotic food category, which must include a specified quantity of beneficial microbial strains, and the fermented category, which is characterized by the microbial transformation process and may or may not include live microorganisms at consumption (178). This differentiation is key to regulatory transparency and consumer classification. Moreover, the sheer heterogeneity of the fermentome, the multitude of metabolites including bioactive peptides, organic acids and EPS, increases the challenge of determining a standard dose of health benefits. Commercial product surveys show substantial variation in the number of microorganisms and concentration of metabolites depending on starter cultures and fermentation conditions employed and demonstrate the importance of standardized characterisation in future nutrition policies (179).

The physiological basis of including fermented foods in preventive nutrition is supported by the growing evidence of their association with reduced risk factors for metabolic diseases. The epidemiological data showed that frequent intake of fermented dairy such as yogurt has been associated with the management of weight, enhanced ability to keep glycemic levels in normal ranges and reduced occurrence of T2D (180). This is attributable to the production of SCFA and bioactive peptides that regulate gut-brain signaling and systemic inflammation. In order to more effectively introduce these findings into the public health strategy, future policies shall be directed toward evidence-based frameworks that consider the aggregate effect of the food matrix and its microbial metabolites. Standardization of labeling to sign about the existence of living cultures and investments in culturally appropriate nutrition education will also be strategy of capitalizing the potential of functional fermented foods to the health of the populace (176).

### 9.2 Role in community nutrition and metabolic disease prevention programs

The integration of functional fermented foods into community nutrition and public health programs offers a biologically plausible and culturally resonant strategy for the prevention of metabolic diseases. The use of fermented foods in community-based intervention and school nutrition is recognizing fermented foods as extremely valuable food items because they can increase the nutrient density and stability of foods, especially in resource-limited settings. As an example, initiatives to include traditional fermented cereals, such as Mahewu, in school feeding programs have been piloted as an inexpensive way to improve the quality of the diet and use locally available products (181). Such traditional ferments are a vital source of essential proteins, vitamins and useful LAB that enhance the overall nutritional outcome of staple-based institutional menus, which may in turn offer protective nutritional benefits to the developmental causes of metabolic syndrome in early-life (10).

Such programs are particularly effective in the case of marginalized populations and food-insecure populations that are completely disrupted by the triple burden of malnutrition, including undernutrition, micronutrient deficiencies, and obesity-related metabolic disorders. Fermented food has the potential to positively regulate the intestinal microbiome related to both weight regulation and systemic inflammation, and this may indicate a specialized role in communities particularly vulnerable to obesity and T2D disease (182). Mechanistic evidence further indicates that the bioactive metabolites and microbial peptides produced during fermentation have antihypertensive properties and insulin-like roles and can help to inhibit the occurrence of hypertension and hyperglycemia in the risk population. Although population-level outcome measures are a domain of future research, these physiological advantages make a solid case of incorporating fermented products into the context of larger preventive nutrition programs to minimize inequality in chronic diseases (10).

One of the most significant benefits of traditional fermented foods in terms of serving the public health in the United Arab Emirates is its inherent accessibility and affordability. These foods are usually made out of local staples with artisanal techniques, which allows local-level production and generation of income with minimal capital input (37). Such social-economic aspect is central to the sustainability, as it enables the ethnic groups to sustain the food sovereignty and enhance the health of the population. Nevertheless, the transition between the household-level production and the implementation of effective community-based programs necessitates the strategic investments into the training, standardization, and the straightforward quality control measures, which are required to achieve safety and consistency (183). Indonesian fermented foods and the African School meal programs is a pilot study to show that with modern safety standards applied to the traditional way of processing, such foods can be scaled to successful institutional use. Innovative community health programs ought to thus mainly focus on the testing of standardized fermented food interventions to affirm their predictable archetype in averting metabolic diseases at population levels (184).

### 9.3 Consumer acceptance, dietary habits, and cultural considerations

The integration of functional fermented foods into public health nutrition strategies depends heavily on consumer acceptance, which is shaped by a complex interplay of sensory attributes, cultural heritage, and perceived health benefits. Sensory characteristics—specifically taste, aroma, and texture—are the primary determinants of consumer preference and repeat purchase behavior. Studies have shown that immediate organoleptic appeal often outweighs health messaging in the decision-making process for everyday dietary choices (185). While fermentation naturally introduces unique sour or pungent notes that can be polarizing, optimizing the fermentation process through specific strain selection and controlled conditions can mitigate sensory aversions and broaden product appeal. For instance, tailoring acidity and texture in plant-based or cereal ferments is essential for gaining acceptance among consumers who may be unaccustomed to traditional, highly acidic profiles (186).

Cultural heritage and traditional knowledge provide a robust foundation for the habitual consumption of fermented foods. In regions where products like kimchi, kefir, natto, and tempeh are staple components of the diet, these foods are not only trusted for their safety and nutritional value but also deeply embedded in culinary legacy (186). Leveraging these existing dietary habits is a strategic advantage for public health initiatives; by innovating within familiar formats, developers can introduce functional enhancements—such as increased concentrations of GABA or bioactive peptides—without triggering “food neophobia” or rejection. Conversely, in populations where fermented foods are less common, unfamiliarity and sensory dislikes, such as pungent odors or unusual textures, remain significant barriers to adoption (95).

Education and targeted communication play a pivotal role in facilitating the integration of these foods into preventive nutrition frameworks for metabolic health. There is a documented awareness gap among consumers, many of whom express interest in the health effects of fermented foods but lack the specific knowledge to differentiate between basic fermented products and clinically validated functional variants (187). Public health education campaigns that highlight the metabolic benefits of fermented bioactives—such as their roles in glycemic control and anti-inflammatory signaling—can shift consumer motivations toward more health-conscious choices. Furthermore, improving regulatory clarity and standardizing health claims will foster consumer trust and support informed decision-making, ultimately promoting the long-term adoption of functional fermented foods as a sustainable dietary strategy for metabolic disease prevention (188).

### 9.4 Safety, regulatory frameworks, and labeling issues

Ensuring the safety and efficacy of functional fermented foods is a cornerstone of their successful implementation in public health nutrition. While fermentation is traditionally used as a preservation method, it is not without inherent safety risks. Potential hazards include the accumulation of chemical contaminants like biogenic amines (e.g., histamine and tyramine), which can cause adverse physiological reactions in sensitive individuals, and biological risks such as mycotoxins or contamination with pathogens like *Listeria monocytogenes* and *Salmonella* (186). Although LAB often inhibit the growth of spoilage and pathogenic organisms through acidification and bacteriocin production, these natural defenses do not eliminate the need for rigorous safety controls and microbial surveillance. Therefore, the use of well-characterized starter cultures with a proven safety record (such as those with “Qualified Presumption of Safety” or QPS status) is critical to minimize risks (95).

Standardization of production and microbial monitoring are essential for both consumer safety and the substantiation of health claims. Inconsistent fermentation conditions can lead to high variability in the concentrations of bioactive metabolites and the viability of probiotic strains, undermining the functional value of the product (4). Precision fermentation technologies, which utilize controlled parameters for pH, temperature, and duration, are increasingly necessary to ensure that target bioactives such as GABA or anti-inflammatory peptides—are present at effective doses throughout the product’s shelf life. Routine testing for contaminants and microbial stability ensures that the product delivers its promised benefits without compromising safety, particularly in foods intended for vulnerable or food-insecure populations (185).

The regulatory landscape for fermented and probiotic foods remains fragmented, presenting significant challenges for international trade and consumer communication. While agencies like the European Food Safety Authority (EFSA) have established strict guidance for the scientific assessment and approval of health claims, other jurisdictions vary in their evidence requirements and labeling standards (188). A major point of confusion for consumers and practitioners alike is the distinction between “fermented foods” and products carrying “probiotic” claims. While all fermented foods are produced via microbial action, only those containing defined, well-characterized live microorganisms with a documented health benefit at a specific dose can be accurately termed probiotics. This distinction is often blurred in marketing, leading to potential misinformation (95).

Inconsistent labeling and the lack of harmonized international standards, such as those discussed within the Codex Alimentarius framework, complicate the integration of fermented foods into formal preventive nutrition strategies. Misleading claims can conflate traditional home-style ferments with clinically validated functional foods, potentially undermining public trust in evidence-based nutrition (189). Achieving a transparent and harmonized regulatory environment, one that mandates safety surveillance and clearly distinguishes between live culture content and therapeutic probiotic claims, is essential to protect public health and support the role of fermented foods in metabolic disease prevention (189).

## 10 Challenges, knowledge gaps, and future research directions

Although there is growing evidence of the metabolic health benefits of functional fermented foods, there are significant gaps and constraints in knowledge that need to be met to translate mechanistic knowledge into evidence-based approaches to public health nutrition. Challenges encountered in the field include severe constraints in the standardization of products, validation of biomarkers, design of clinical trials, the design of personalized interventions, and integration of molecular information at the systems level. To address these challenges, interdisciplinary research in food science, microbiology, clinical nutrition and computational biology fields will be necessary. One of the most crucial issues is the absence of standardization of the composition of fermented foods and biomolecule analysis. There is also a lot of heterogeneity among fermented products, components and processing, among fermentations and fermentation-derived metabolites, making it difficult to attribute disease-specific dietary exposure to reproducible health effects (19). To limit this variability, harmonized reporting standards comprising minimal compositional data, including microbial taxonomy and viability (measured in quantitative live counts), complete metabolite panels (including short-chain fatty acids, EPS, bioactive peptides and biogenic amines) and detailed raw-material metadata (of substrate properties and fermentation conditions) is urgently needed. Standardized analytical pipelines, such as shotgun metagenomics to profile microbes and both targeted and untargeted metabolomics to quantify biologic active compounds, and the creation of analytical reference materials are needed to permit significant comparison of studies, to support regulated evaluation, and enable meta-analyses (190). In addition, common description of safety profiles, dose range, and target population characteristics are required to support both rational design of the clinical trial and truth-driven public health intervention. Another important knowledge gap is the process of identifying and authenticating mechanistic biomarkers predicting metabolic outcomes. Although a variety of candidate biomarkers have been suggested, such as fermentation-produced metabolites, e.g., SCFAs, EPS, bacteriocins, modified polyphenols, and bioactive peptides; no single candidate biomarker has yet been prospectively tested and confirmed a predictor of clinically meaningful metabolic outcomes in human beings (191). There are often microbial signatures of temporary colonization or of changes in community diversity and functional resilience after fermented food consumption, and which may be intermediate biomarkers. Mechanistically, host-response markers and cardiometabolic benefits in preclinical models have been linked to NRF2-related anti-inflammatory mediator prototypes and cytoprotective signaling pathways. Fermentation-derived biomolecules, including bioactive peptides, short-chain fatty acids, and transformed polyphenols, may exert protective metabolic effects through activation of antioxidant signaling pathways such as nuclear factor erythroid 2–related factor 2 (Nrf2) pathway (192). However, prospective validation of individual responses and long-term metabolic disease prognosis using specific biomarker panels is absent, impairing its value for individualized intervention plans. The primary evidence gap is large-scale, long-term clinical trials; existing human studies are small, short-term, heterogeneous in product characterization, and have inconsistent outcomes and poorly defined mechanistic sampling, limiting causal clarity and generalizability (193, 194).

Chronic metabolic illnesses manifest over the years to decades, but most fermented food trials measure surrogate endpoints in the short run but not long-term microbiome regulation, safety profiles or disease incidence and progression. This large methodological heterogeneity among trials in product description, doses, blinding, choice of endpoints, and mechanistic tests further restricts the viability of meta-analysis and synthesis of evidence. Although fermented foods have been shown to have metabolic and anti-inflammatory advantages in many animal models, limited clinical translation has yet to be performed in humans in a consistent methodology (193). To define the relationship between transient microbiome and metabolic alterations leading to long-lasting metabolic disease risk reduction, to define optimal dose and safety profiles, and to describe interindividual heterogeneity in prolonged treatment responses, long-term, adequately powered randomized controlled trials with standard characterization of product are required. Microbiome-directed and personalized nutrition are a prospective and uncharted territory. The presence of substantial interindividual variability regarding response to the intervention of fermented food indicates the possibility of stratifying based on the microbiome composition at baseline, metabolic phenotype, or genetic background (19). Theoretically, a tailored therapy model based on the correlation of individual host deficiencies or functional gaps in the microbiome with the attributes of particular fermented food products could maximize therapeutic success, such as the use of live microbial strains, metabolic phenotypes, and substrate composition (195). Strategies of fermented food synbiotics merging with complementary prebiotics or custom-engineered probiotic strains could rise rising the colonization resistance and personalize the gains. Nevertheless, there are no validated clinical algorithms that are reliable in the allocation of individuals to a particular fermented food intervention, with predictable metabolic outcomes, and the future creation of such frameworks will require trials, in large cohorts of hundreds of individuals, with the rich phenotypic characterization and machine learning strategies (95). Lastly, there is a need to incorporate multi-omics technologies and systems biology methods of explaining the causal mechanisms that connect the composition of fermented food and its effects on metabolic health. Longitudinal studies involving microbial metagenomics and metatranscriptomics, intensive fecal and plasma metabolomics to measure SCFAs, polyphenol metabolic products, and bioactive peptide synthesis, and long-term inflammatory and metabolic pathways transcriptomic or proteomic profiling have been demanded in the field (196). Future research should prioritize the identification of host and microbial stratifiers that influence individual responses to fermented food interventions. Factors such as baseline gut microbiota composition, including the abundance of key taxa such as *Akkermansia muciniphila* and *Bifidobacterium* spp., may affect the production and utilization of fermentation-derived biomolecules. In addition, host genetic polymorphisms in metabolite-sensing receptors such as free fatty acid receptor 2 (FFAR2) and free fatty acid receptor 3 (FFAR3), habitual dietary patterns, and baseline metabolic phenotype may contribute to differential responsiveness. Stratification based on these host and microbial factors could facilitate the development of precision nutrition strategies using functional fermented foods for targeted prevention and management of metabolic diseases.

## 11 Conclusion

Functional fermented foods represent a advanced delivery system for a diverse array of microbial metabolites, transformed polyphenols, and bioactive peptides that collectively modulate host physiology. The primary biomolecular mechanisms involve the regulation of key metabolic signaling pathways, such as AMPK/PGC1α and PI3K/Akt, alongside the microbial production of SCFAs like acetate, which enhance energy balance and attenuate systemic inflammation. These components act as an “extended microbiome,” bridging dietary inputs with host metabolic regulation through intricate gut microbiota interactions that shift microbial ecology toward beneficial taxa like *Bacteroides* and *Alistipes*. The preventive importance of these foods in metabolic disease is increasingly supported by evidence showing their ability to mitigate obesity, dyslipidemia, hepatic steatosis, and insulin resistance. By improving glucose tolerance and lipid profiles, fermented matrices offer a potent non-pharmacological approach to managing chronic metabolic disorders. From a public health perspective, fermented foods provide a scalable, culturally adaptable, and cost-effective strategy for population-level health promotion. However, the future potential of this field lies in the transition toward precision nutrition, where designer fermented systems and postbiotics are tailored to individual microbiome signatures to optimize therapeutic outcomes. To fully realize these implications, future research must prioritize standardized manufacturing frameworks and large-scale human clinical trials to translate preclinical mechanistic insights into validated public health recommendations. Ultimately, integrating fermentation science with microbiome diagnostics will be essential for addressing the global burden of metabolic disease.
